# Therapeutic efficacy of biofeedback pelvic floor muscle exercise in women with dysfunctional voiding

**DOI:** 10.1038/s41598-021-93283-9

**Published:** 2021-07-02

**Authors:** Ching-Hsiang Chiang, Yuan-Hong Jiang, Hann-Chorng Kuo

**Affiliations:** Department of Urology, Hualien Tzu Chi Hospital, Buddhist Tzu Chi Medical Foundation, and Tzu Chi University, No. 707, Chung-Yang Rd., Sec. 3, Hualien, Taiwan, ROC

**Keywords:** Urology, Urethra

## Abstract

Dysfunctional voiding (DV), a voiding dysfunction due to hyperactivity of the external urethral sphincter or pelvic floor muscles leading involuntary intermittent contractions during voiding, is not uncommon in neurologically normal women with lower urinary tract symptoms (LUTS). We aimed to investigate the therapeutic efficacy of biofeedback pelvic floor muscle training (PFMT) in female patients with DV and to identify the therapeutic efficacy. Thirty-one patients diagnosed with DV. All participates completed the 3-month biofeedback PFMT program, which was conducted by one experienced physiotherapist. At 3 months after treatment, the assessment of treatment outcomes included global response assessment (GRA), and the changes of clinical symptoms, quality of life index, and uroflowmetry parameters. 25 (80.6%) patients had successful outcomes (GRA ≥ 2), and clinical symptoms and quality of life index significantly improved after PFMT. Additionally, uroflowmetry parameters including maximum flow rate, voided volume, voiding efficiency, total bladder capacity, voiding time, and time to maximum flow rate significantly improved after PFMT treatment. Patients with the history of recurrent urinary tract infection in recent 1 year were found to have unsatisfied therapeutic outcomes. In conclusion, biofeedback PFMT is effective in female patients with DV with significant improvements in clinical symptoms, quality of life, and uroflowmetry parameters. The history of urinary tract infection in recent 1 year is a negative predictor of successful outcome.

## Introduction

Voiding dysfunction caused by anatomical or functional bladder outlet obstruction (BOO) among women has received increasing attention recently^1^. Inappropriate activities of the urethral sphincter and/or pelvic floor muscles (PFMs) during voiding can cause functional BOO, and in the patients with intact neurologic functions it is called dysfunctional voiding (DV)^2^. The voiding flow pattern of DV is intermittent and/or fluctuating, and it is one of the common nonstructural and/or non-neurogenic voiding dysfunctions in women^3^. DV prevalence rates of between 2 and 35% in women with LUTS has been reported^4^. DV leads to not only bothersome storage lower urinary tract symptoms (LUTS), such as urinary frequency and urgency, but also voiding difficulty, urinary retention, and a higher risk of recurrent urinary tract infection (UTI) that significantly impact the patients’ quality of life^5–8^.

Biofeedback pelvic floor muscle training (PFMT) is a conservative treatment that includes the assessment of pelvic floor strength and the functional use of PFMs. Such treatment increases the contractility and holding strength, coordination, velocity, and endurance of the PFMs via programmed voluntary muscle contraction and relaxation practice^9^. Several types of exercises have been used for PFMT, including passive, active-assisted, active-resisted, or simple contraction exercises. It can be performed with electrostimulation, biofeedback, and vaginal cones^10,11^. These positive effects are beneficial for patient with stress urinary incontinence, overactive bladder, chronic pelvic muscle pain and pediatric DV^12–18^.

Theoretically, PFMT could correct both the hypertonicity and poor relaxation of PFMs and modulate bladder sensation and detrusor overactivity. Only few data have indicated the positive outcomes of PFMT in female adults with DV, with a response rate of 51–83%^19,20^. Therefore, we aimed to investigate the therapeutic efficacy of biofeedback PFMT in female patients with DV and to identify the therapeutic efficacy.

## Methods

### Patients

**Figure 1:**
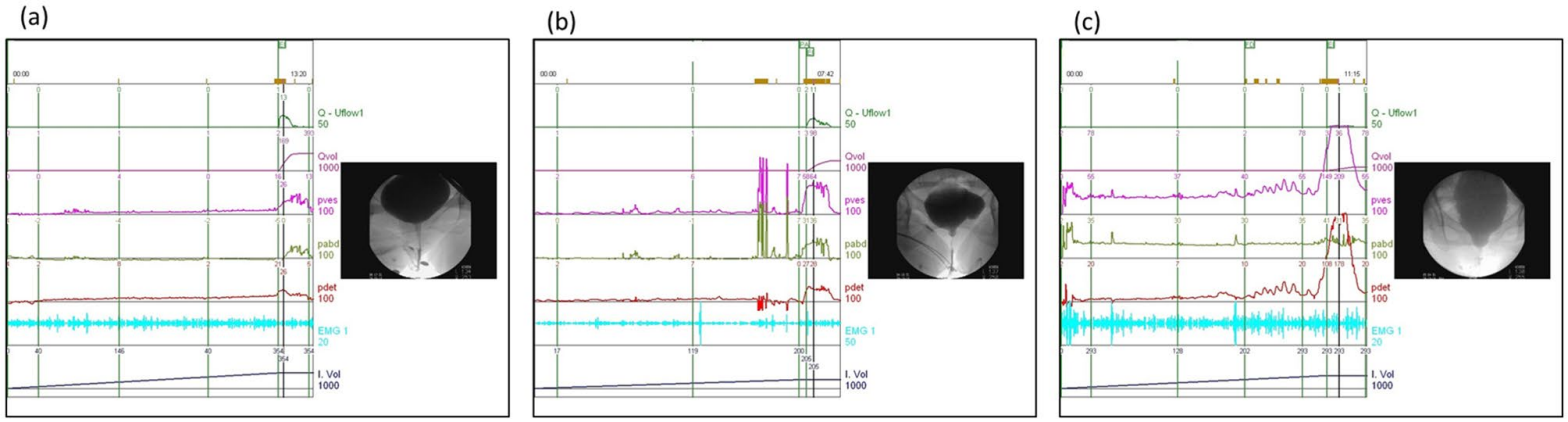
Videourodynamic characteristics of (**a**) Normal, (**b**) DV and (**c**) severe DV appearance.

Female DV patients who underwent biofeedback PFMT were retrospectively reviewed from 2017 to 2019. The Ethics Committee of Buddhist Tzu Chi General Hospital had approved the study. (IRB Number: IRB109-095-B). All methods were performed in accordance with the relevant guidelines and regulations. Patients were informed about the study rationale and procedures; written informed consent was obtained from all patients prior to enrollment and treatment. The diagnosis of DV was based on VUDS and according to International Continence Society terminology^2^. In urodynamically normal appearance, it included low voiding pressure with smooth urinary flow pattern, relaxed electromyography activity on external urethral sphincter, and open bladder neck and urethra picture on real-time fluoroscopy (Fig. 1a). Patients with a high voiding pressure with a low and/or intermittent urinary flow during voiding, a picture of open bladder neck but pelvic floor muscle or membranous urethra level narrowing on real-time fluoroscopy and increased external urethral sphincter electromyography activities were diagnosed with DV (Fig. 1b,c).

All cases had tried medication for initial LUTS before the PFMT, such as non-selective selective alpha blocker, antispasmodic agent and anti-muscarinic/beta 3-adrenergic receptor agonist depending on patient’s condition and medical compliance. The failure of conventional medical would be confirmed if one of following criteria existed: (1) the patient reported no subjective improvement according to GRA < 1, (2) impaired flow picture on uroflowmetry (maximal flow rate < 10 ml/s with obstructive flow pattern), or (3) clean intermittent catherization/urethral catheterization was needed due to retention. The patients received the biofeedback PFMT program after the conventional medical treatment failed.

All eligible patients’ clinical history had been reviewed from medical chart, and brief neurologic examination had been performed in the first interview (12 cranial nerves examination, bulbocavernosus reflex, anal reflex, and somatic sensory of dermatomes and peripheral limbs). Renal and bladder sonography was completed to survey upper urinary tract deterioration causing by high bladder pressure and high residual volumes. Cystoscopy was performed to exclude the diagnosis of urethral structure. Patients who had traceable history of neurologic deficit, remarkable abnormality on neurologic examination, the other anatomical BOO, active urinary tract infection/vaginitis/pelvic inflammatory disease during the program, genital prolapse, interstitial cystitis, the history of pelvic surgery for urinary incontinence or cancer, and radiation therapy were excluded from the study.

### Training protocol

**Figure 2:**
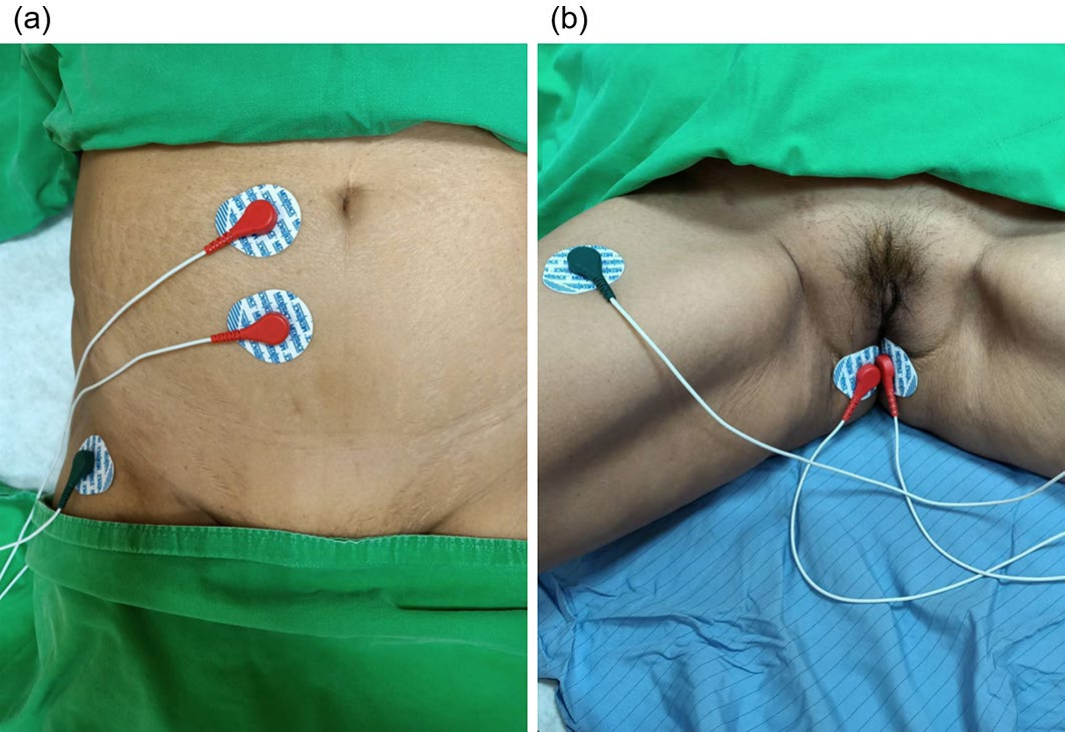
The application of surface 2-channel electromyography (EMG) electrodes over (**a**) abdominal rectus muscles and (**b**) perineum.

The biofeedback PFMT program was conducted by a single experienced physiotherapist, with total six office training course in a private therapy room during a 3-month treatment period. The interval between the office courses was 2–3 weeks. A brief history interview was completed at the first visit. The initial assessment was performed using the Modified Oxford Grading System to evaluate the strength of the PFM via vaginal palpation^21^. Surface 2-channel electromyography (EMG, *Pathway*^*®*^* MR-25 Dual channel surface EMG system, The Prometheus Group, Dover, New Hampshire, USA*) electrodes were applied at the perineum to collect the resting EMG signals of the PFM and abdominal rectus muscles (Fig. 2).

**Figure 3:**
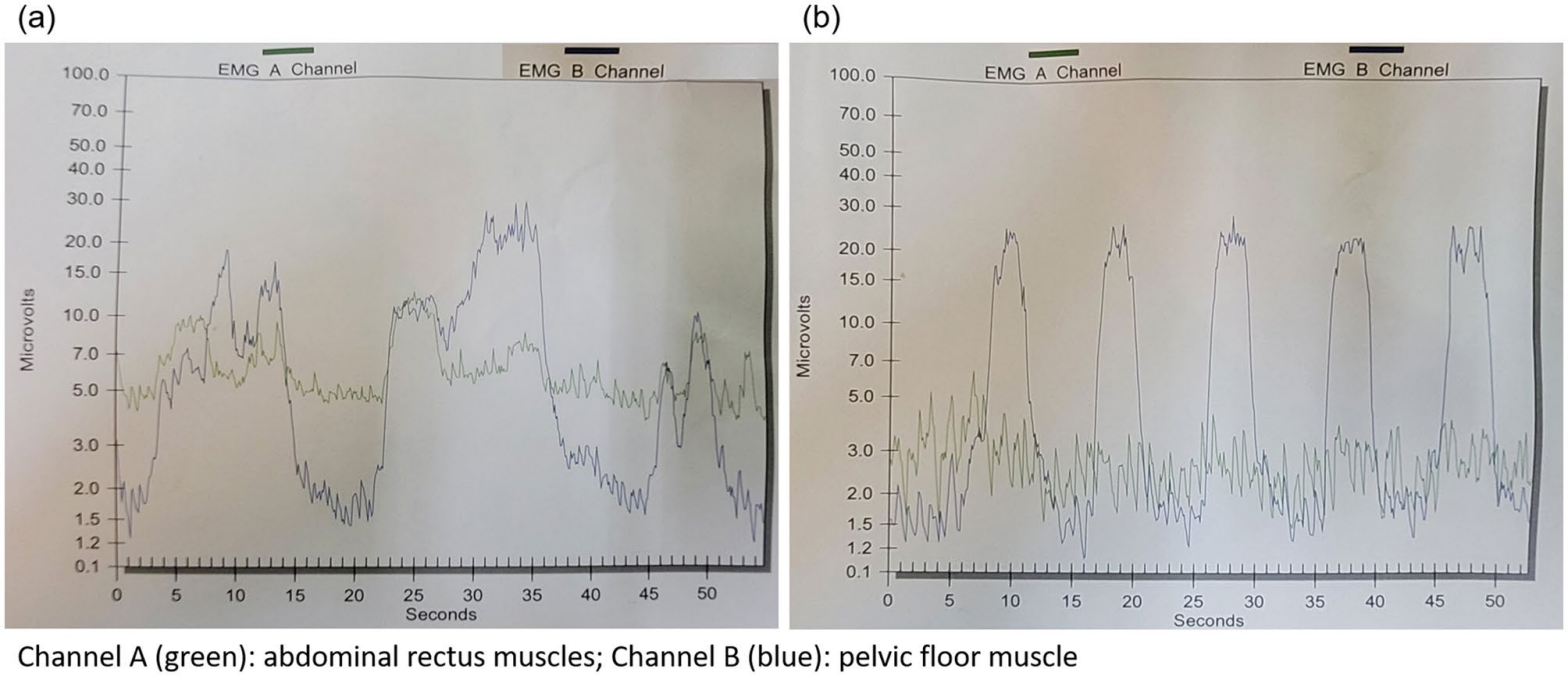
The example of electromyography on (**a**) inadequate PFM isolation, contraction and poor relaxation at the first time of office visiting; (**b**) An adequate picture of EMG which indicated well PFM isolation and relaxation after biofeedback PFMT at the 6th time of office visiting.

The PFM therapeutic modalities included the followings^22^. The initial steps were PFM isolation and discrimination training. The patients were instructed to strengthen the PFM under real-time visual EMG monitor, and verbal instruction and reinforcement were provided to ensure that the patients voluntarily contracted the correct isolated PFM group without involvement of the accessory muscles of the buttocks, legs, or abdomen. Such step focused on the elimination of accessory muscle substitution via the identification and modulation of the associated muscle groups as well as enhancing sensory awareness and release variations to maximize conscious control of muscle contraction. The next parts were PFM strengthening and endurance training: the patients were instructed to appropriately tighten the PFM in several different positions (supine, sitting, and standing). The physiotherapist placed 2 fingers into the vagina as digital exam and asked the patient to perform pelvic floor contraction, just like to withhold voiding as she normally did. Adequate contraction would be determined according to modified Oxford scale > 1 (0 = no contraction, 1 = flicker, 2 = weak contraction, 3 = moderate, 4 = good and 5 = strong) on digital exam finding. The endurance was set to 3–5 s in the beginning and was increased up to 10 s to enhance and maintain PFM motor recruitment. It was followed by the relaxation of PFM to baseline EMG level within 2 s to adequately relax the PFMs (Fig. 3).

The interval between each pelvic floor contraction was at least twice as long as that of endurance of contraction, which was referred to as down-training, a procedure inhibiting hypertonic muscle activity to lower elevated resting tone. There were 10 repetitions in one session, and 3 sessions in a course were recommended initially. It could be up to 20 repetitions in each session and up to 6 sessions according to the patient’s ability. In the home program, the patients practiced at least 3 sessions daily. Telephone interview had been performed weekly to assess the compliance of home program and resolve the problem about the practice from the patient.

### Outcome assessment

The primary treatment outcome was assessed by the information regarding Global Response Assessment (GRA; categorized into − 3, − 2, − 1, 0, 1, 2, 3, indicating markedly worse to markedly improved status) of satisfaction at 3 months treatment period. Successful outcome was defined as GRA ≥ 2 (moderate and marked improved). The secondary treatment outcomes comprised the changes of symptom scores, and uroflowmetry parameters at 3 months after treatment. The symptom scores for assessment included total International Prostate Symptom Score (IPSS-T), IPSS-S (storage subscore), IPSS-V (voiding subscore), Patient Perception of Bladder Condition (PPBC) score, and quality of life (QoL) index, which had been validated for female LUTS in published articles^23,24^. Uroflowmetry parameters included total bladder capacity (TBC), voided volume (VV), postvoid residual volume (PVR), maximum flow rate (Qmax), corrected Qmax (cQmax, defined as Qmax/√TBC), voiding efficiency (VE, defined as VV/TBC), average flow rate (Qave), flow time of voiding (T-flow), voiding time (T-voiding), time to Qmax during voiding (T-Qmax) and flow curve shape. The normal values of Qmax was defined as 12 mL/s or 15 mL/s for TBC of 150 mL or 200 mL^25,26^. The PVR was measured by the bladder sonography. The baseline clinical characteristics and urodynamic parameters were also analyzed to identify the predictive factors of treatment outcomes.

### Statistical analysis

The continuous variables were presented as means with standard deviations. Wilcoxon sign rank test was used to distinguish the difference of variables between baseline and post-treatment status. Wilcoxon rank sum test was used for statistical comparisons of variables in between-subgroup analysis. The baseline clinical characteristics and urodynamic parameters were analyzed using the Wilcoxon rank sum test for independent samples. Logistic binary regression was used to analyze the predictive factors of successful outcomes. A *p*-value of < 0.05 was considered to indicate statistical significance. All statistical analyses were performed in a personal computer using the Statistical Package for the Social Sciences software for Windows (version 25.0; SPSS Chicago, IL, the USA).

## Results

A total of 31 female patients, with the mean age 55.6 ± 15.5 (range 18–77) years, completed the biofeedback PFMT course and follow-up investigations. Table 1 showed the changes of clinical symptoms and uroflowmetry parameters. After treatment, 25 (80.6%) patients had successful outcomes (GRA ≥ 2). All symptoms scores including IPSS-T, IPSS-S, IPSS-V, PPBC, and QoL index significantly improved after biofeedback PFMT. Additionally, uroflowmetry parameters including Qmax, proportion of low Qmax, cQmax, VV, TBC, Qave, T-voiding, and T-Qmax significantly improved. There were 3 types of flow curve appearing in the cohort (bell-shaped, staccato-shaped and plateau-shaped). Before the PFMT, 6.5% of patients revealed non-obstructive (bell-shaped) flow curve, and 93.5% in obstructive pattern (Staccato-shaped: 58.1%; Plateau-shaped: 35.5%). After the 3-month biofeedback PFMT, it showed less obstructive picture revealed (29.0%, Staccato-shaped: 19.4%; Plateau-shaped: 9.7%). There was no patient had remarkable upper tract deterioration as hydronephrosis before the program, and no case who needed CIC or catheterization before and after the PFMT program.

**Table 1:** Changes of clinical symptoms and uroflowmetry parameters in female DV patients after PFMT at 3-month follow-up.

	Baseline	Follow-up	p value
**Clinical symptoms**			
IPSS-S	8.4 ± 4.4	3.2 ± 3.0	< 0.001
IPSS-V	11.3 ± 6.2	4.4 ± 4.3	< 0.001
IPSS-T	19.7 ± 8.2	7.6 ± 6.6	< 0.001
IPSS-QoL	5.1 ± 0.9	2.1 ± 1.5	< 0.001
PPBC	5.0 ± 0.9	2.1 ± 1.5	< 0.001
**Uroflowmetry parameters**			
Qmax	13.1 ± 7.3	20.2 ± 7.4	< 0.001
Low Qmax^a^	20 (64.5%)	3 (9.7%)	< 0.001
cQmax	0.8 ± 0.3	1.1 ± 0.3	< 0.001
VV	237.3 ± 170.0	326.3 ± 139.4	0.003
PVR	27.9 ± 34.8	28.6 ± 23.9	0.916
TBC	265.3 ± 187.7	346.6 ± 137.2	0.010
VE	0.89 ± 0.12	0.93 ± 0.86	0.062
Qave	6.8 ± 3.3	11.6 ± 4.6	< 0.001
T-flow	37.0 ± 22.9	31.5 ± 14.5	0.173
T-voiding	42.5 ± 29.9	30.3 ± 12.9	0.009
T-Qmax	13.3 ± 13.3	6.7 ± 2.6	0.012
**Uroflowmetry flow curve shape**			
Bell-shaped	2 (6.5%)	22 (71.0%)	< 0.001
Staccato-shaped	18 (58.1%)	6 (19.4%)	
Plateau-shaped	11 (35.5%)	3(9.7%)	

*IPSS* International Prostate Symptom Score, *IPSS-S* IPSS storage subscore, *IPSS-V* IPSS voiding subscore, *IPSS-T* Total IPSS, *IPSS-QoL* IPSS quality of life score, *PPBC* Patient Perception of Bladder Condition, *Qmax* maximum flow rate, *cQmax *corrected maximum flow rate, *VV* voided volume, *PVR* postvoid residual volume, *TBC* total bladder capacity, *VE* voiding efficacy, *Qave* average flow rate, *T-flow* flow time, *T-voiding* voiding time, *T-Qmax* time to Qmax.

^a^< 12 mL/s or < 15 mL/s for total bladder capacity of 150 mL or 200 mL.

The patients with successful outcomes had significantly higher percentage of recurrent UTI history in recent 1 year, and lower IPSS-V in baseline clinical characteristics, and higher cQmax in baseline uroflowmetry than those with failure outcomes (Table 2). After treatment, the patients with successful outcomes had significantly improved in Qmax, VV, TBC, VE, cQmax, Qave, T-voiding, and T-Qmax. However, the patients with failure outcomes did not have significant changes in uroflowmetry parameters except Qave.

**Table 2:** Clinical characteristics and uroflowmetry profiles in patients with different treatment outcomes.

Baseline clinical characteristics	Success (N = 25)	Failure (N = 6)	p value
Age	58.16 ± 13.42	45.00 ± 20.37	0.061
Recurrent UTI	2 (8%)	3 (50%)	0.038
Constipation	10 (60.0%)	5 (83.3%)	0.083
Diabetes Mellitus	2 (8.0%)	1 (16.7%)	0.488
Body mass index	23.6 ± 4.1	24.2 ± 5.3	0.763
Psychologic disabilities^a^	3 (33.3%)	2 (12.0%)	0.241
Menopause	13 (52.0%)	3 (50.0%)	1.000
**Vaginal delivery**	22 (88.0%)	4 (66.7%)	0.241
Parity	2.0 ± 1.2	1.3 ± 1.0	0.218
**Cesarean delivery**	2 (8.0%)	0 (0.0%)	1.000
Parity	0.2 ± 0.6	0.0 ± 0.0	0.490
Strength of pelvic floor muscle^b^	2.7 ± 0.9	2.8 ± 1.0	0.794
IPSS-S	8.1 ± 4.6	9.5 ± 4.0	0.502
IPSS-V	10.2 ± 6.2	15.8 ± 4.2	0.043
IPSS-T	18.3 ± 8.3	25.3 ± 6.9	0.059
IPSS-QoL	5.1 ± 0.9	5.0 ± 0.6	0.767
PPBC	5.1 ± 0.8	4.7 ± 1.5	0.540
**Uroflowmetry parameters**			
**Qmax**			
Baseline	13.8 ± 7.7	10.1 ± 4.7	0.276
△	7.8 ± 8.1*	4.4 ± 5.8	0.338
**cQmax**			
Baseline	0.9 ± 0.3	0.6 ± 0.1	0.017
△	0.3 ± 0.4*	0.2 ± 0.3	0.598
**VV**			
Baseline	237.4 ± 161.8	236.8 ± 218.3	0.994
△	108.7 ± 145.1*	6.8 ± 172.6	0.146
**PVR**			
Baseline	22.9 ± 25.2	49.0 ± 59.5	0.337
△	3.9 ± 26.1	− 12.7 ± 63.6	0.557
**TBC**			
Baseline	260.3 ± 171.0	285.8 ± 265.2	0.771
△	102.2 ± 146.8*	− 5.8 ± 220.1	0.153
**VE**			
Baseline	0.91 ± 0.10	0.83 ± 0.17	0.197
△	0.04 ± 0.01*	0.01 ± 0.11	0.481
**Qave**			
Baseline	6.5 ± 3.2	7.9 ± 3.4	0.369
△	5.1 ± 4.7*	5.1 ± 4.7*	0.976
**T-flow**			
Baseline	38.4 ± 24.1	31.7 ± 17.5	0.531
△	− 6.2 ± 25.5	3.7 ± 11.2	0.364
**T-voiding**			
Baseline	43.9 ± 32.6	36.8 ± 16.0	0.610
△	− 12.6 ± 28.2*	− 3.0 ± 6.9	0.132
**T-Qmax**			
Baseline	14.8 ± 14.5	7.2 ± 2.6	0.219
△	− 8.0 ± 13.9*	2.0 ± 2.4	0.092
**Uroflowmetry flow curve**			
**Obstructive**			
Baseline	23(92%)	6(100%)	0.474
△	17(68%)*	3(50%)	0.208

*UTI* urinary tract infection, *IPSS* International Prostate Symptom Score, *IPSS-S* IPSS storage subscore, *IPSS-V* IPSS voiding subscore, *IPSS-T* Total IPSS, *IPSS-QoL* IPSS quality of life score, *PPBC* Patient Perception of Bladder Condition, *Qmax* maximum flow rate, *cQmax *corrected maximum flow rate, *VV* voided volume, *PVR* postvoid residual volume, *TBC* total bladder capacity, *VE* voiding efficacy, *Qave* average flow rate, *T-flow* flow time, *T-voiding* voiding time, *T-Qmax* time to Qmax.

△: Changes of values/case number/proportion after PFMT.

*p value < 0.05 versus baseline value.

^a^Defined as known psychologic disease with definite diagnosis from psychiatrist and psychotic medication use.

^b^Modified Oxford Grading System score.

Univariate and multivariate logistic regression analysis revealed that the history of recurrent UTI in recent 1 year is the only independent negative predictive factor of successful outcomes of biofeedback PFMT (OR 0.01, p = 0.042) (Table 3). DV female patients with the history of recurrent UTI were associated with poor treatment outcomes of PFMT. No case had traceable past history of learned habitual disturbance in childhood, incontinence, polycystic ovaries or inflammation disease as following: vaginitis or pelvic inflammatory disease, proctitis, hemorrhoidal crisis (severe bleeding, incarceration, thrombosis).

**Table 3:** Univariate and multivariate logistic regression analysis for the predictive factors of successful outcomes of PFMT.

Factors	Univariate		Multivariate	
	Odds ratio (95% CI)	p value	Odds ratio (95% CI)	p value
Recurrent UTI	0.09 (0.01–0.75)	0.026*	0.01 (0.00–0.83)	0.042*
IPSS-V	0.82 (0.67–1.01)	0.065	0.71 (0.49–1.05)	0.086
cQmax	15.29 (0.45–561.28)	0.128	45.33 (0.09–23,643.67)	0.232

*UTI* urinary tract infection, *IPSS-V* International Prostate Symptom Score voiding subscore, *cQmax *corrected maximum flow rate.

*p value < 0.05.

## Discussion

This study revealed that biofeedback PFMT is effective in more than 80% of female DV patients with significant improvements in clinical symptoms, QoL, and uroflowmetry parameters. The patients with successful treatment outcomes had significant improvements in not only clinical symptoms but also uroflowmetry parameters, which indicated the clinical efficacy of biofeedback PFMT. Additionally, the history of urinary tract infection in recent 1 year is a negative predictor of successful outcome of biofeedback PFMT in female DV patients. Different treatment strategy other than biofeedback PFMT might be considered in these patients.

Dysfunctional voiding is a voiding dysfunction due to functional BOO, usually causes bothersome LUTS and intractable in clinical practice^2^. In women with intact neurological function, voiding is initiated when a voluntary signal is sent from the suprapontine centers to activate the pontine micturition center to trigger voiding reflex. This action is mediated through spinal centers and involves the relaxation of the external urethral sphincter muscle and the PFM, followed by the contraction of the bladder via parasympathetic innervation^27^. Functional BOO due to PFM spasticity (nonrelaxing or hypertonicity of external urethral sphincter/PFM) results in an incomplete evacuation of urine^28^, which is considered a learned habitual disturbance occurring in response to urgency or pelvic discomfort caused by inappropriate toilet training or some adverse events (such as infection, inflammation, trauma, or irritation) during childhood or early adult life^29–31^. Accordingly, non-invasive biofeedback PFMT is aimed to correct the disturbance and resume the normal function of PFM during voiding.

In a previous study of the VUDS analysis, women with DV showed higher rates of detrusor overactivity and bladder hypersensitivity, lower TBC and VV, lower Qmax and VE, and higher PVR than normal patients^4^. If the patients hold urine back during urgency episodes, a long-term increase in the external urethral sphincter activity caused by the reflex inhibitory response to the occurrence of detrusor overactivity might result in the spasticity of the external urethral sphincter. As a long-term effect of urgency caused by detrusor overactivity, the spasticity of the external urethral sphincter results in DV. High voiding pressure status of BOO due to DV might induce detrusor overactivity or bladder sensory impairment. Detrusor overactivity or sensory urgency dysfunction might be the primary etiology of DV or secondary to DV, and it plays an important role in the pathophysiology and development of DV^32^. In this study, DV patients also had both bothersome storage and voiding symptoms, low Qmax, low TBC, and poor QoL.

Biofeedback PFMT has been widely used to treat overactive bladder, urge and postpartum incontinence in adult patients as well as DV in pediatric patients^17,33,34^. In terms of storage symptom improvement, biofeedback PFMT can modulate overactive bladder syndrome by increasing urethral pressure, leading to the inhibition of the sacral preganglionic innervation to the bladder through the guarding reflex^35^. PFM contraction can also stimulate the sympathetic nerve fibers of the internal urethral sphincter, thereby causing a decrease in detrusor muscle pressure^36,37^. Our study revealed that both storage and voiding symptoms improved after biofeedback PFMT with the increases in bladder capacity and urinary flow rate. It indicated that the restoration of normal PFM function might not only reduce the bladder outlet dysfunction but also the bladder dysfunction in DV.

As for the amelioration of voiding problem in DV, biofeedback PFMT may help resolve functional BOO. During PFM exercise, the surface EMG provided a summary of the activities of the PFM (the external anal and urethral sphincters), and most patients with DV were found to have nonrelaxing and hypertonic urethral sphincter EMG activity. Either the striated external urethral sphincter or the PFM muscle or both can contribute to DV and can be differentiated based on the VUDS findings of women with normal neurological function. In this study, the benefits of biofeedback PFMT included not only the elimination of voiding symptoms but also the remarkable improvements in uroflowmetry parameters (including Qmax, cQmax, Qave, VV, TBC, T-voiding, and T-Qmax). The comprehensive PFMT program focused more on relaxation, which helps in achieving the extinction of hypertonicity of the EUS and PFM as well as a more rapid PFM relaxation during the voiding stage.

In women with recurrent UTI, the incidence of lower urinary tract dysfunctions was high, and DV was diagnosed in 25% patients^38^. Numerous risk factors and etiologies of recurrent UTI have been identified, including anatomical characteristics, hormonal status, pregnancy, hygiene, and lifestyle habits^39^. Whenever possible, to correct the predisposing factors of recurrent UTI is needed in the treatment. A randomized study reported by Minardi et al. revealed that biofeedback PFMT could not only improve on both emptying and storage symptoms but also significantly decreased the prevalence of UTIs in female DV patient, while the relapse of symptoms occurred after training stopped^40^. In the present study, the DV patients with the history of recurrent UTI within 1 year were associated with poor treatment outcomes of biofeedback PFMT. To provide the treatment of biofeedback PFMT might not be sufficient in these patients, and more compressive management for recurrent UTI and different treatment strategy for DV might be needed.

There were several limitations in this study. First, this was a retrospective analysis with a small case number in a single center, and the follow-up course was not scheduled uniformly. Second, we did not enroll the other DV patients as control/placebo group for comparison. A large comprehensive prospective cohort study to validate the efficacy of biofeedback PFMT in women with DV is needed.

## Conclusion

Biofeedback PFMT is effective in more than 80% of female patients with DV with significant improvements in clinical symptoms, quality of life, and uroflowmetry parameters. The patients with the history of recurrent UTI in recent 1 year were associated with poor treatment outcomes. Different treatment strategy other than biofeedback PFMT might be considered in these patients.

## Data Availability

To protect patient privacy and comply with relevant regulations, identified data are unavailable. Requests from qualified researchers with appropriate ethics board approvals and relevant data use agreements for de-identified data will be available.

## References

[1] Meier K, Padmanabhan P (2016). Female bladder outlet obstruction: An update on diagnosis and management. Curr. Opin. Urol..

[2] Haylen BT, de Ridder D, Freeman RM (2010). An International Urogynecological Association (IUGA)/International Continence Society (ICS) joint report on the terminology for female pelvic floor dysfunction. Neurourol. Urodyn..

[3] Carlson KV, Fiske J, Nitti VW (2000). Value of routine evaluation of the voiding phase when performing urodynamic testing in women with lower urinary tract symptoms. J. Urol..

[4] Peng C-H, Chen S-F, Kuo H-C (2017). Videourodynamic analysis of the urethral sphincter overactivity and the poor relaxing pelvic floor muscles in women with voiding dysfunction. Neurourol. Urodyn..

[5] Gunnarsson M, Mattiasson A (1999). Female stress, urge, and mixed urinary incontinence are associated with a chronic and progressive pelvic floor/vaginal neuromuscular disorder: An investigation of 317 healthy and incontinent women using vaginal surface electromyography. Neurourol. Urodyn..

[6] Kuo H-C (2005). Videourodynamic characteristics and lower urinary tract symptoms of female bladder outlet obstruction. Urology.

[7] Sinha S (2011). Dysfunctional voiding: A review of the terminology, presentation, evaluation and management in children and adults. Indian J. Urol..

[8] Artibani W, Cerruto MA (2014). Dysfunctional voiding. Curr. Opin. Urol..

[9] Bertotto A, Schvartzman R, Uchôa S, Wender MCO (2017). Effect of electromyographic biofeedback as an add-on to pelvic floor muscle exercises on neuromuscular outcomes and quality of life in postmenopausal women with stress urinary incontinence: A randomized controlled trial. Neurourol. Urodyn..

[10] Schreiner L, dos Santos TG, de Souza ABA, Nygaard CC, Silva Filho IG (2013). Electrical stimulation for urinary incontinence in women: A systematic review. Int. Braz. J. Urol..

[11] Moroni RM, Magnani PS, Haddad JM, de Castro RA, Brito LGO (2016). Conservative treatment of stress urinary incontinence: A systematic review with meta-analysis of randomized controlled trials. Rev. Bras. Ginecol. Obstet..

[12] Burns PA, Pranikoff K, Nochajski TH, Hadley EC, Levy KJ, Ory MG (1993). A comparison of effectiveness of biofeedback and pelvic muscle exercise treatment of stress incontinence in older community-dwelling women. J. Gerontol..

[13] Wyndaele JJ, Hoekx L, Vermandel A (1997). Bladder biofeedback for the treatment of refractory sensory urgency in adults. Eur. Urol..

[14] Burgio KL, Locher JL, Goode PS (1998). Behavioral vs drug treatment for urge urinary incontinence in older women: A randomized controlled trial. JAMA.

[15] Elser DM, Wyman JF, McClish DK, Robinson D, Fantl JA, Bump RC (1999). The effect of bladder training, pelvic floor muscle training, or combination training on urodynamic parameters in women with urinary incontinence. Continence Program for Women Research Group. Neurourol. Urodyn..

[16] Bø K, Talseth T, Holme I (1999). Single blind, randomised controlled trial of pelvic floor exercises, electrical stimulation, vaginal cones, and no treatment in management of genuine stress incontinence in women. BMJ.

[17] Kulaksizoğlu H, Akand M, Çakmakçi E, Gül M, Seçkin B (2015). Effectiveness of pelvic floor muscle training on symptoms and uroflowmetry parameters in female patients with overactive bladder. Turk. J. Med. Sci..

[18] Voorham JC (2017). The effect of EMG biofeedback assisted pelvic floor muscle therapy on symptoms of the overactive bladder syndrome in women: A randomized controlled trial. Neurourol. Urodyn..

[19] Hoebeke P, Vande Walle J, Theunis M, De Paepe H, Oosterlinck W, Renson C (1996). Outpatient pelvic-floor therapy in girls with daytime incontinence and dysfunctional voiding. Urology.

[20] Liaw Y-M, Kuo H-C (2007). Biofeedback pelvic floor muscle training for voiding dysfunction and overactive bladder. Incont. Pelvic Floor Dysfunct..

[21] Pedraza R, Nieto J, Ibarra S, Haas EM (2014). Pelvic muscle rehabilitation: A standardized protocol for pelvic floor dysfunction. Adv. Urol..

[22] Laycock J, Jerwood D (2001). Pelvic floor muscle assessment: The PERFECT Scheme. Physiotherapy.

[23] Okamura K, Nojiri Y, Osuga Y, Tange C (2009). Psychometric analysis of international prostate symptom score for female lower urinary tract symptoms. Urology.

[24] Coyne KS, Matza LS, Kopp Z, Abrams P (2006). The validation of the patient perception of bladder condition (PPBC): A single-item global measure for patients with overactive bladder. Eur. Urol..

[25] Massey JA, Abrams PH (1988). Obstructed voiding in the female. Br. J. Urol..

[26] Costantini E, Mearini E, Pajoncini C, Biscotto S, Bini V, Porena M (2003). Uroflowmetry in female voiding disturbances. Neurourol. Urodyn..

[27] de Groat WC, Yoshimura N, Vodušek DB, Boller F (2015). Chapter 5—Anatomy and physiology of the lower urinary tract. Handbook of Clinical Neurology.

[28] Kuo TLC, Ng LG, Chapple CR (2015). Pelvic floor spasm as a cause of voiding dysfunction. Curr. Opin. Urol..

[29] Groutz A, Blaivas JG, Pies C, Sassone AM (2001). Learned voiding dysfunction (non-neurogenic, neurogenic bladder) among adults. Neurourol. Urodyn..

[30] Abrams P, Cardozo L, Fall M (2003). The standardisation of terminology in lower urinary tract function: Report from the standardisation sub-committee of the International Continence Society. Urology.

[31] Carlson KV, Rome S, Nitti VW (2001). Dysfunctional voiding in women. J. Urol..

[32] Chen Y-C, Kuo H-C (2014). Clinical and video urodynamic characteristics of adult women with dysfunctional voiding. J. Formos Med. Assoc..

[33] Kahyaoglu Sut H, Balkanli KP (2016). Effect of pelvic floor muscle exercise on pelvic floor muscle activity and voiding functions during pregnancy and the postpartum period. Neurourol. Urodyn..

[34] de Jong TPVM, Klijn AJ, Vijverberg MAW, de Kort LM, van Empelen R, Schoenmakers MAGC (2007). Effect of biofeedback training on paradoxical pelvic floor movement in children with dysfunctional voiding. Urology.

[35] Chai TC, Steers WD (1997). Neurophysiology of micturition and continence in women. Int. Urogynecol. J. Pelvic Floor Dysfunct..

[36] Shafik A, Shafik IA (2003). Overactive bladder inhibition in response to pelvic floor muscle exercises. World J. Urol..

[37] Greer JA, Smith AL, Arya LA (2012). Pelvic floor muscle training for urgency urinary incontinence in women: A systematic review. Int. Urogynecol. J..

[38] Lee P-J, Kuo H-C (2020). High incidence of lower urinary tract dysfunction in women with recurrent urinary tract infections. Low Urin. Tract Symptoms..

[39] Guglietta A (2017). Recurrent urinary tract infections in women: Risk factors, etiology, pathogenesis and prophylaxis. Future Microbiol..

[40] Minardi D, d’Anzeo G, Parri G (2010). The role of uroflowmetry biofeedback and biofeedback training of the pelvic floor muscles in the treatment of recurrent urinary tract infections in women with dysfunctional voiding: A randomized controlled prospective study. Urology.

